# Diagnostic performance of adenosine deaminase for abdominal tuberculosis: A systematic review and meta-analysis

**DOI:** 10.3389/fpubh.2022.938544

**Published:** 2022-09-21

**Authors:** Ruixi Zhou, Xia Qiu, Junjie Ying, Yan Yue, Tiechao Ruan, Luting Yu, Qian Liu, Xuemei Sun, Shaopu Wang, Yi Qu, Xihong Li, Dezhi Mu

**Affiliations:** ^1^Department of Pediatrics, West China Second University Hospital, Sichuan University, Chengdu, China; ^2^Key Laboratory of Birth Defects and Related Diseases of Women and Children, Sichuan University, Ministry of Education, Chengdu, China

**Keywords:** adenosine deaminase, abdominal tuberculosis, ascites, meta-analysis, diagnostic value

## Abstract

**Background and aim:**

Abdominal tuberculosis (TB) is a common type of extrapulmonary TB with an insidious onset and non-specific symptoms. Adenosine deaminase (ADA) levels increase rapidly in the early stages of abdominal TB. However, it remains unclear whether ADA serves as a diagnostic marker for abdominal TB.

**Methods:**

We performed a systematic literature search for relevant articles published in PubMed, Web of Science, Cochrane Library, and Embase up to April 2022. First, we used the Quality Assessment of Diagnostic Accuracy Studies tool-2 (QUADAS-2), to evaluate the quality of the included articles. Bivariate and hierarchical summary receiver operating characteristic (HSROC) models were then utilized to analyze pooled sensitivity, specificity, positive likelihood ratio (PLR), negative likelihood ratio (NLR), diagnostic odds ratio (DOR) and area under the receiver operating characteristic curve (AUROC). In addition, we explored a subgroup analysis for potential heterogeneity and publication bias among the included literature.

**Results:**

Twenty-four articles (3,044 participants, 3,044 samples) which met the eligibility criteria were included in this study. The pooled sensitivity and specificity of ADA for abdominal TB detection were 93% [95% confidence interval (CI): 0.89–0.95] and 95% (95% CI: 0.93–0.96), respectively. PLR and NLR were 18.6 (95% CI: 14.0–24.6) and 0.08 (95% CI: 0.05–0.12), respectively. DOR and AUROC were 236 (95% CI: 134–415) and 0.98 (95% CI: 0.96–0.99), respectively. Furthermore, no heterogeneity or publication bias was found.

**Conclusions:**

Our meta-analysis found ADA to be of excellent diagnostic value for abdominal TB and could be used as an auxiliary diagnostic tool.

**Systematic review registration:**

https://www.crd.york.ac.uk/prospero/, identifier: CRD42022297931.

## Introduction

Tuberculosis (TB) is one of the most serious global health conditions, with high prevalence and mortality rates (1); millions of new cases are reported worldwide, and ~1.2 million people die from TB each year, particularly in high-burden countries such as India and Central Africa (1–3). Abdominal TB accounts for 6.1% of all extrapulmonary TB cases, and the incidence of abdominal TB in pulmonary TB patients is 10–30% (4, 5). *Mycobacterium tuberculosis* (*Mtb*) can spread through the blood and lymph to the abdominal cavity or *via* the digestive tract and adjacent organs causing abdominal TB (6, 7), which can be further classified into mesenteric, peritoneal, intestinal, and visceral TB (5, 8–10). Ascites is one of the most common clinical manifestations of abdominal TB (11). Abdominal TB has an insidious course, which can delay diagnosis and treatment, and result in increased disease severity and mortality (6, 12, 13). In order to reduce severity, mortality, and morbidity, it is important to make a timely diagnosis and institute effective treatment.

At present, the golden standard in clinical diagnosis of abdominal TB is still laparoscopic pathological biopsy and/or culture of *Mtb* with ascites (5). However, the high cost and invasiveness of laparoscopy make it impossible to be used routinely in clinics. Furthermore, adverse events (3%) and mortality (0.04%) have been reported in laparoscopic detection (5). The culture of *Mtb* has a low positive rate (25 to 36% in ascitic fluid) and takes up to 8 weeks to provide a result (5, 14). Thus, both biopsy and culture are impractical for the early diagnosis in patients with abdominal TB. Additionally, there are other examination methods available in clinics, e.g., blood tests, biochemical examinations, GeneXpert MTB/RIF assay, and imaging methods. Blood tests show signs of chronic inflammation but are non-specific (6). Biochemical examination of ascites may suggest exudation, but this may be indistinguishable from diseases such as cirrhosis, which often coexist with abdominal TB (15). The GeneXpert MTB/RIF assay, a heminested real-time polymerase chain reaction method, has high diagnostic power for pulmonary samples but low sensitivity for extrapulmonary samples (16, 17). Imaging methods, such as ultrasound and computed tomography, can only assist in paracentesis and tissue biopsy but cannot provide a definitive diagnosis (18, 19). Therefore, developing a detection method which is rapid, efficient, and economical would be conducive to both the early detection, and timely and effective treatment of abdominal TB.

Adenosine deaminase (ADA) is an enzyme that degrades immunosuppressive signals due to adenosine and plays an important regulatory role in immune homeostasis (20). While infecting the patient, *Mtb* can cause an imbalance of host innate and adaptive immune homeostasis resulting in TB (21). Recently, ADA levels were found to be significantly upregulated in a variety of TB cavity effusions, suggesting that ADA could be used as a marker for diagnosing TB (22–26). ADA was reported to have a high value in diagnosing abdominal TB, even in patients with low immunity caused by human immunodeficiency virus (HIV) infection (27, 28). As a test which requires minimally invasive sampling, ADA also has high clinical applicability. However, in some low-burden countries, such as the USA (sensitivity: 58.8%, specificity: 95.4%) and South Korea (sensitivity: 82%, specificity: 79%), ADA diagnostic performance is unsatisfactory (29, 30). In addition, ADA has been reported to have different sensitivities and specificities for diagnosing TB when using different cut-off values (31–33). Therefore, finding an optimal cut-off value could improve the availability of the ADA test for abdominal TB screening. Here, we performed a systematic review and meta-analysis of published ADA results to explore its overall diagnostic value in abdominal TB.

## Methods

### Literature search

This study was based on PRISMA-DTA statement published in 2018 and registered in PROSPERO (CRD42022297931) (34). Two independent reviewers searched and retrieved original English research articles in PubMed, Web of Science, Cochrane Library, and Embase, since each database's creation until April 2022. The following Medical Subject Heading keywords in the text, title, and abstract of published literature were used to identify relevant articles: “tuberculosis,” “tuberculous,” “abdominal,” “peritonitis,” “ascites,” “peritoneal,” “TBP,” “intestinal,” “mesenteric lymph node,” “mesenteric lymph nodal,” “extra-pulmonary,” “adenosine deaminase,” and “ADA.” References (forward citations) and citation lists (backward citations) of the relevant literature were also consulted to find as many available articles as possible. Two independent reviewers examined all available literature to discuss and resolve any discrepancies.

### Study selection

Research articles were included according to the following inclusion criteria: (i) cases of abdominal TB and non-TB controls; (ii) ADA level in ascites as the index test; (iii) clinical diagnosis, bacteriology, or histopathology as reference standards for abdominal TB; and (iv) sensitivity and specificity of ADA as primary outcomes, with more than five participants in each study. Reviews, abstracts, comments, case reports, papers published in a language other than English, and animal experiments were excluded. Two independent reviewers evaluated the articles to eliminate eligibility bias according to the above requirements. Consensus was reached through discussion and scientific persuasion, and qualified articles were included in our study for further processing.

### Data extraction

We extracted the following data from each article: country/region, TB burden (World Health Organization adjustment) (35); study design type; abdominal TB category; reference standard; number of participants (abdominal TB and non-TB controls); method; sample type; ADA cut-off values; and ADA sensitivity, specificity, true positive (TP), false positive (FP), false negative (FN), and true negative (TN) rates. Data extraction was independently evaluated and cross-checked by two reviewers, and consensus was reached through discussion.

### Quality assessment

In accordance with QUADAS-2, two independent reviewers assessed the quality of the included articles. RevMan (version 5.3) was used for the analysis (36).

### Data analysis

The data analyses were conducted using the HSROC, and bivariate models were constructed with the “metandi” package in Stata (version 14.0) (37, 38). Pooled sensitivity, specificity, positive likelihood ratio (PLR), negative likelihood ratio (NLR), diagnostic odds ratio (DOR), HSROC curve, and area under the summary receiver operating characteristic curve (AUROC) were calculated with a confidence interval (CI) of 95% (39). The underlying factors of potential heterogeneity were grouped and analyzed by meta-regression analysis. Four subgroups were considered: TB-burden country/region (high or low), study design type (case-control or not), disease category (TB ascites or not), and different cut-offs of ADA (≥40 IU/L or not) (40). Publication bias was assessed by Deeks' funnel plot asymmetry test (41). Statistical significance was set at *p* < 0.05.

## Results

### Search results

**Figure 1:**
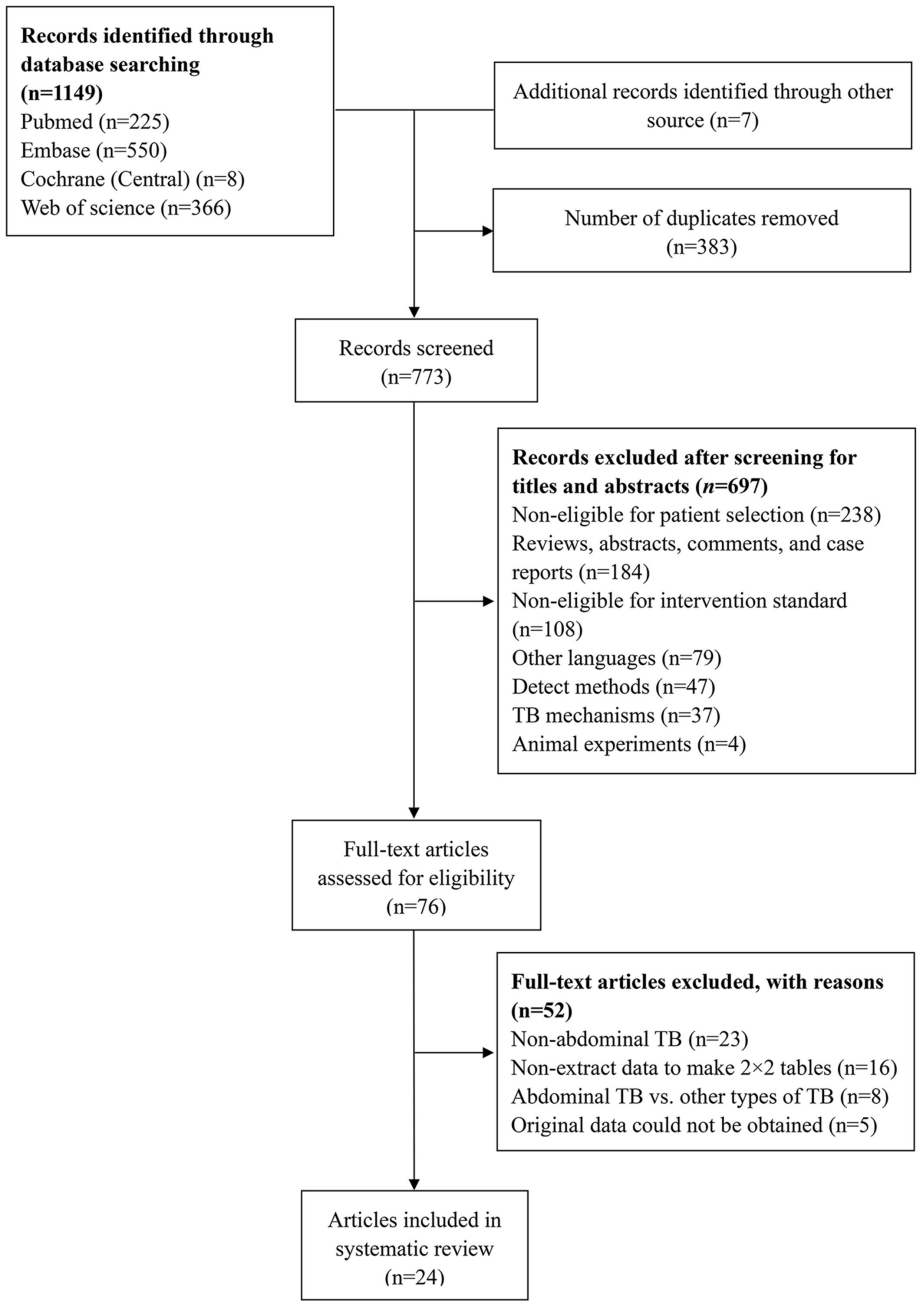
Flow chart of the process of the identified articles regarding ADA and abdominal TB.

Among 1,156 studies retrieved, 383 were considered duplicates (the same article) and therefore, excluded. Upon preliminary screening, 697 studies were excluded: 238 studies were ineligible based on the patient selection criteria (cirrhosis, abdominal tumor, Crohn's disease, etc.); 184 studies consisted of reviews, abstracts, comments, and case reports; 108 studies were ineligible based on the intervention criteria (interferon-γ, new tuberculosis vaccine, T-SPOT.TB, etc.); 79 studies consisted of non-English publications (Chinese, Spanish, Japanese, etc.); 47 used other detection methods (computed tomography, polymerase chain reaction, immunochromatographic assay, etc.); 37 studies focused on TB mechanisms (signaling pathway, cell death, immune response, etc.); and 4 studies included animal experiments (rats, mice, etc.). The full text of remaining articles was reviewed; 24 of the 76 articles were included in the meta-analysis (29–33, 42–60) (Figure 1).

### Main characteristics

The main characteristics of 24 studies were listed in Tables 1, 2 (29–33, 42–60). Data were collected from 11 countries (five low-burden and six high-burden) between 1989 and 2021. A total of 3,044 participants' data (837 patients with abdominal TB and 2,207 participants without TB) were analyzed. Sub-categories of abdominal TB included peritoneal TB, tuberculous peritonitis, and TB ascites. The reference standards for abdominal TB met the following criteria: clinical diagnosis, bacteriology, and histopathology (61, 62). The ADA index was calculated from samples of ascitic fluid according to the methods of Slaats, Giusti et al. as required by our inclusion criteria. The ADA cut-off value to support the diagnosis of abdominal TB was 7–41.1 IU/L. ADA sensitivity, specificity, TP, FP, TN, and FN were also extracted. The weight of abdominal TB was determined by the number of participants in each literature.

**Table 1:** Main characteristics of included studies.

**Reference**	**Country/Region**	**TB burden**	**Study design**	**Category**	**Participants**		**Reference standard**	**Weight of abdominal TB**
					**Abdominal TB**	**non-TB control**		
Dahale et al. (42)	India	High	Case-control	Peritoneal TB	78	208	B + C + H	9.31% (78/837)
Sun et al. (43)	China	High	Cohort	Tuberculous peritonitis	132	147	B + C + H	15.8% (132/837)
He et al. (30)	China	High	Cross-sectional	Tuberculous peritonitis	73	135	B + C + H	8.72% (73/837)
Kumabe et al. (31)	Japan	Low	Cohort	Tuberculous peritonitis	8	173	B + H, culture of pleural effusion, urine, and sputum	0.96% (8/837)
Liu et al. (33)	China	High	Cross-sectional	Tuberculous peritonitis	115	76	B + C	13.74% (115/837)
Lee et al. (29)	South Korea	Low	Cross-sectional	Tuberculous peritonitis	45	29	B + C + H	5.38% (45/837)
Ali et al. (44)	Bangladesh	High	Cross-sectional	Tuberculous peritonitis	24	6	C + H	2.87% (24/837)
Hallur et al. (45)	India	High	Cross-sectional	Peritoneal TB	37	50	B + C + H	4.42% (37/837)
Kang et al. (46)	South Korea	Low	Cross-sectional	Tuberculous peritonitis	27	25	B + H	3.23% (27/837)
Liao et al. (47)	China Taiwan	Low	Cohort	Tuberculous peritonitis	6	211	B + C + H	0.72% (6/837)
Saleh et al. (48)	Egypt	Low	Cross-sectional	Tuberculous peritonitis	14 (14 HIV-positive)	27 (27 HIV-positive)	B + C	1.67% (14/837)
Hong et al. (49)	South Korea	Low	Cross-sectional	Tuberculous peritonitis	41	19	B + C + H	4.90% (41/837)
Gupta et al. (50)	India	High	Cross-sectional	TB ascites	36	72	B + C + H, sputum smear	4.30% (36/837)
Sharma et al. (51)	India	High	Cross-sectional	TB ascites	31	88	B + H, sputum smear	3.70% (31/837)
Burgess et al. (52)	South Africa	High	Cross-sectional	Tuberculous peritonitis	18 (5 HIV-positive)	160	B + C + H, sputum smear or culture	2.15% (18/837)
Sathar et al. (53)	South Africa	High	Cross-sectional	Tuberculous peritonitis	23	22	C + H	2.75% (23/837)
Hillebrand et al. (30)	The United States	Low	Case-control	Tuberculous peritonitis	17	351	B + H	2.03% (17/837)
Brant et al. (54)	Brazil	High	Cross-sectional	Tuberculous peritonitis	8	36	B +H	0.96% (8/837)
Sathar et al. (55)	South Africa	High	Cross-sectional	Tuberculous peritonitis	29 (2 HIV-positive)	53	B + H	3.46% (29/837)
Fernandez-Rodriguez et al. (56)	Spain	Low	Cross-sectional	Peritoneal TB	12	96	B + H	2.03% (17/837)
Ribera et al. (57)	Spain	Low	Cross-sectional	Tuberculous peritonitis	16 (4 HIV-positive)	70 (7 HIV-positive)	B + H	1.43% (12/837)
Bhargava et al. (58)	India	High	Cross-sectional	Peritoneal TB	17	70	B + C + H, sputum smear	1.91% (16/837)
Dwivedi et al. (59)	India	High	Cross-sectional	Tuberculous peritonitis	19	30	B + C + H, culture of sputum	2.03% (17/837)
Voigt et al. (60)	South Africa	High	Cohort	Tuberculous peritonitis	11	53	B + H, sputum smear	2.27% (19/837)

B, bacteriology of ascites; C, clinical diagnosis; H, histopathology; HIV, human immunodeficiency virus; TB, tuberculosis.

**Table 2:** Baseline data regarding ADA of included studies.

**Reference**	**ADA**			**Sensitivity (%)**	**Specificity (%)**	**TP**	**FP**	**FN**	**TN**
	**Assay method**	**Samples**	**Cut-off value (IU/L)**						
Dahale et al. (42)	Slaats	Ascites	41.1	95	93	74	15	4	193
Sun et al. (43)	Giusti	Ascites	21	83.3	95.2	110	7	22	140
He et al. (30)	Peroxidase	Ascites	24.06	90	96.77	66	4	7	131
Kumabe et al. (31)	No available	Ascites	40	100	96	8	7	0	166
Liu et al. (33)	Giusti	Ascites	31.5	89.6	92.1	103	6	12	70
Lee et al. (29)	No available	Ascites	21	82	79	37	6	8	23
Ali et al. (44)	No available	Ascites	24	87.5	83.33	21	1	3	5
Hallur et al. (45)	Modified Giusti	Ascites	36	91.9	88	34	6	3	44
Kang et al. (46)	No available	Ascites	21	92	94.4	25	1	2	24
Liao et al. (47)	Slaats	Ascites	27	100	93.3	6	14	0	197
Saleh et al. (48)	Giusti	Ascites	35	100	92.6	14	2	0	25
Hong et al. (49)	No available	Ascites	30	89	82	36	2	5	16
Gupta et al. (50)	Guisti and Galanti	Ascites	40	100	96	36	3	0	69
Sharma et al. (51)	Giusti	Ascites	37	96.8	94.3	30	5	1	83
Burgess et al. (52)	Giusti	Ascites	30	94	92	17	13	1	147
Sathar et al. (53)	Kinetic enzyme-coupled	Ascites	30	96	100	22	0	1	22
Hillebrand et al. (30)	Enzymology	Ascites	7	58.8	95.4	10	16	7	335
Brant et al. (54)	Giusti	Ascites	30	100	92	8	3	0	33
Sathar et al. (55)	Spectrophotometry	Ascites	30	93	96	27	2	2	51
Fernandez-Rodriguez et al. (56)	Slaats	Ascites	32	83.3	100	10	0	2	96
Ribera et al. (57)	Giusti	Ascites	40	100	97	16	2	0	68
Bhargava et al. (58)	Giusti	Ascites	36	100	97	16	2	0	68
Dwivedi et al. (59)	Giusti	Ascites	33	100	97.1	17	2	0	68
Voigt et al. (60)	Spectrophotometry	Ascites	32.3	100	96.6	19	1	0	29

FN, false negative; FP, false positive; TN, true negative; TP, true positive.

### Quality of the included studies

**Figure 2:**
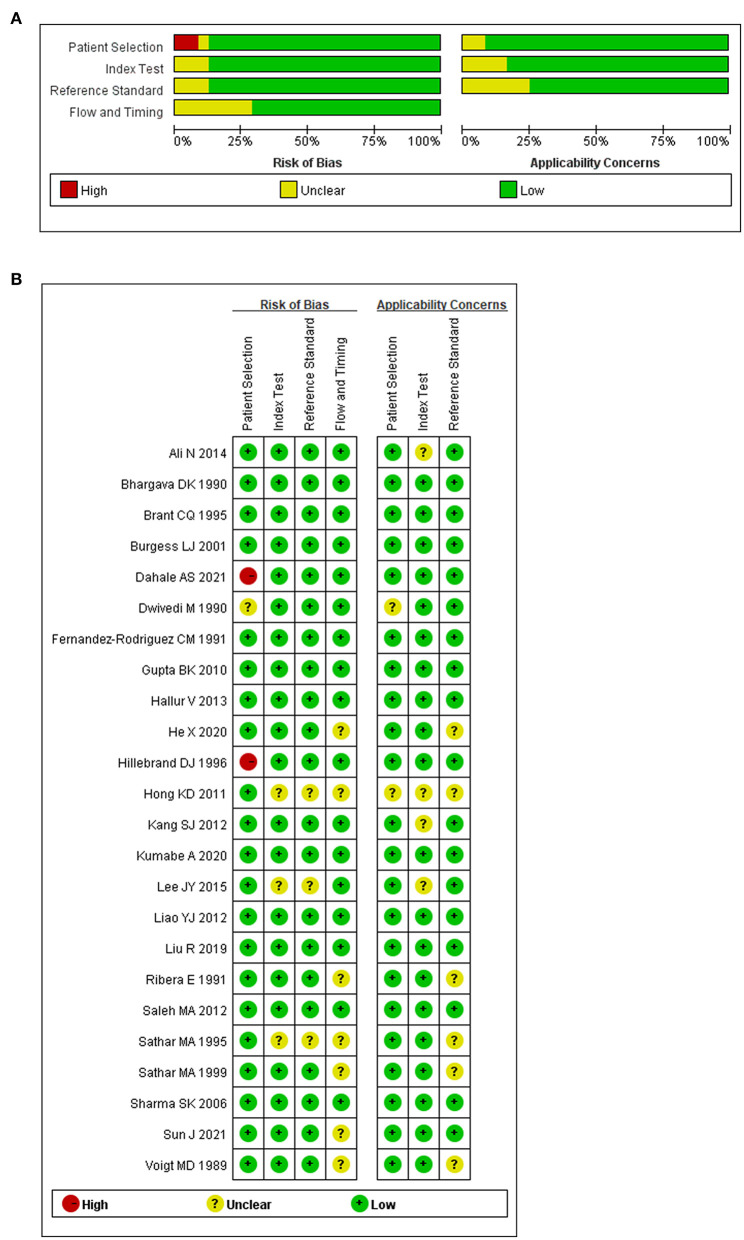
Methodological quality regarding ADA and abdominal TB. **(A)** Graph of risk of bias and applicability concerns. **(B)** Summary of risk bias and applicability concerns.

To avoid bias and ambiguity, the methodological quality of the included studies was evaluated by the four aspects through QUADAS-2 (Figures 2A,B). In patient selection bias, two high-risk articles came from a case-control study (30, 42) and one unclear article lacked an inclusion period (59). In the index test bias, three unclear articles did not report the blinding method (29, 49, 53). The same three unclear articles also did not report the reference standard blinding method (29, 49, 53). Regarding flow and timing bias, seven unclear articles showed that participants accepted different reference standards (30, 42, 43, 49, 55, 57, 60). Generally, the overall quality of the included articles was good.

### Summary statistics

**Figure 3:**
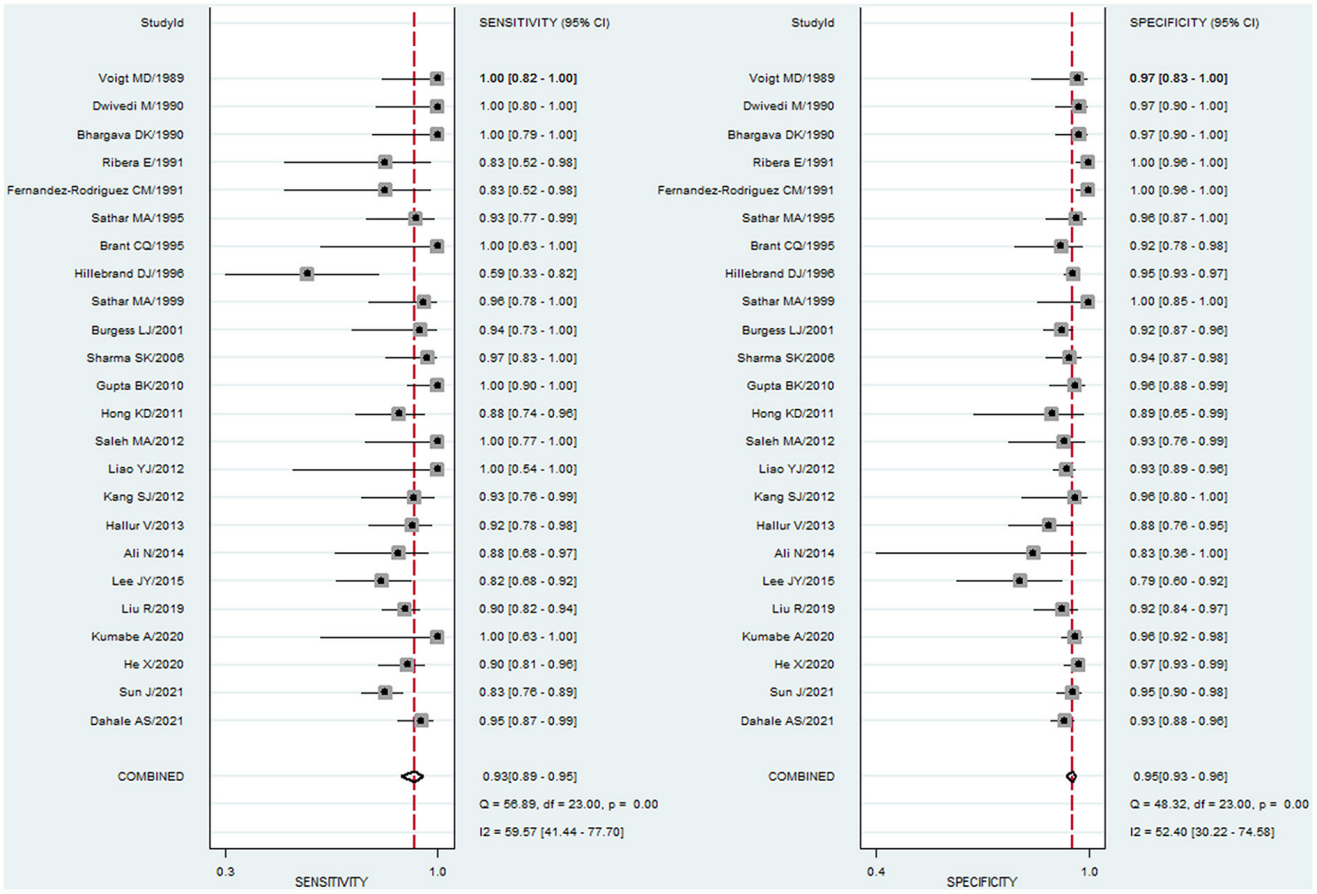
Paired forest plots of pooled sensitivity and specificity of ADA for the diagnosis of abdominal TB. Sensitivity and specificity in each study were represented by squares, and 95% confidence intervals were represented by horizontal bars.

**Figure 4:**
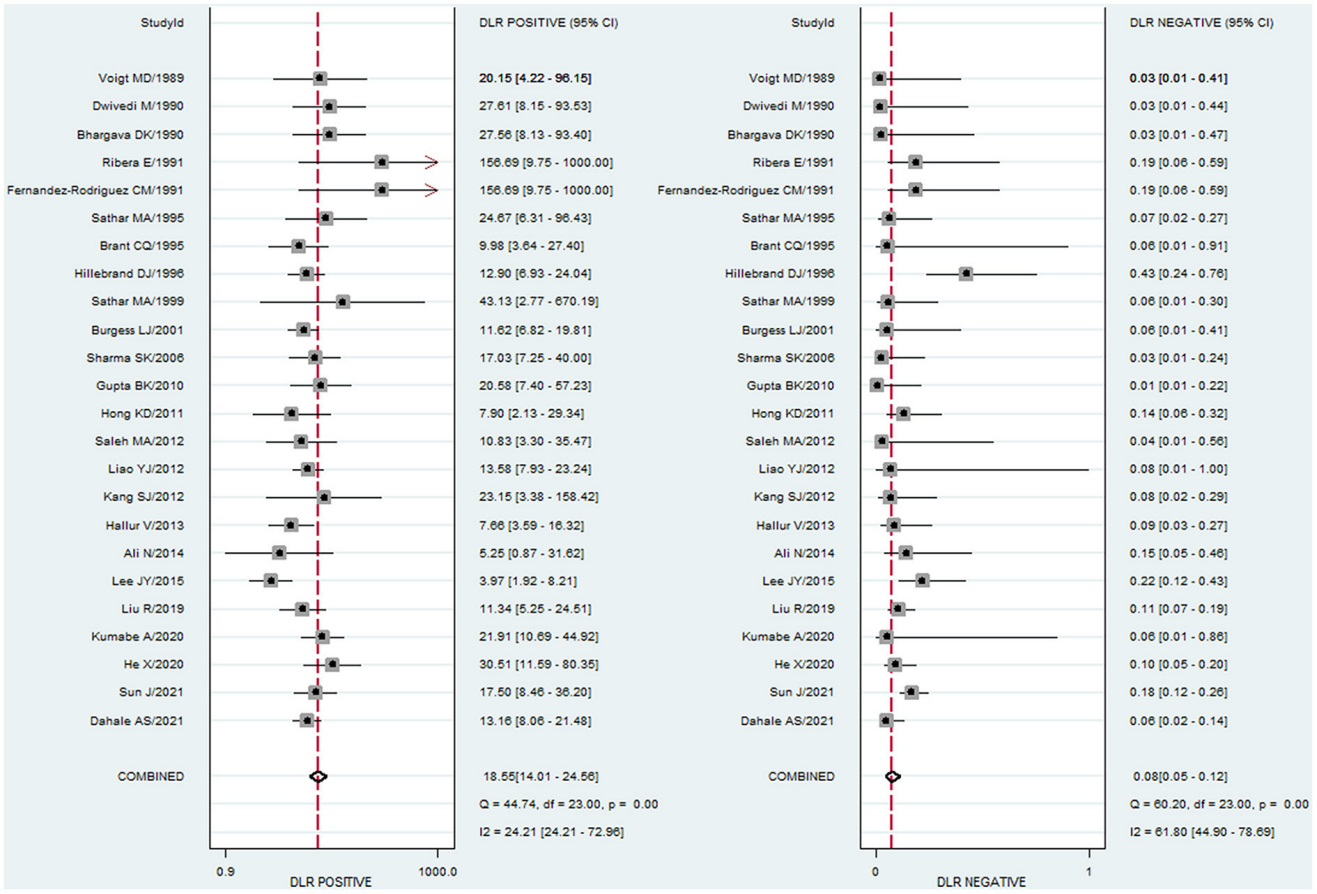
Paired forest plots of combined PLR and NLR of ADA for the diagnosis of abdominal TB. PLR and NLR in each study were represented by squares, and 95% confidence intervals were represented by horizontal bars.

**Figure 5:**
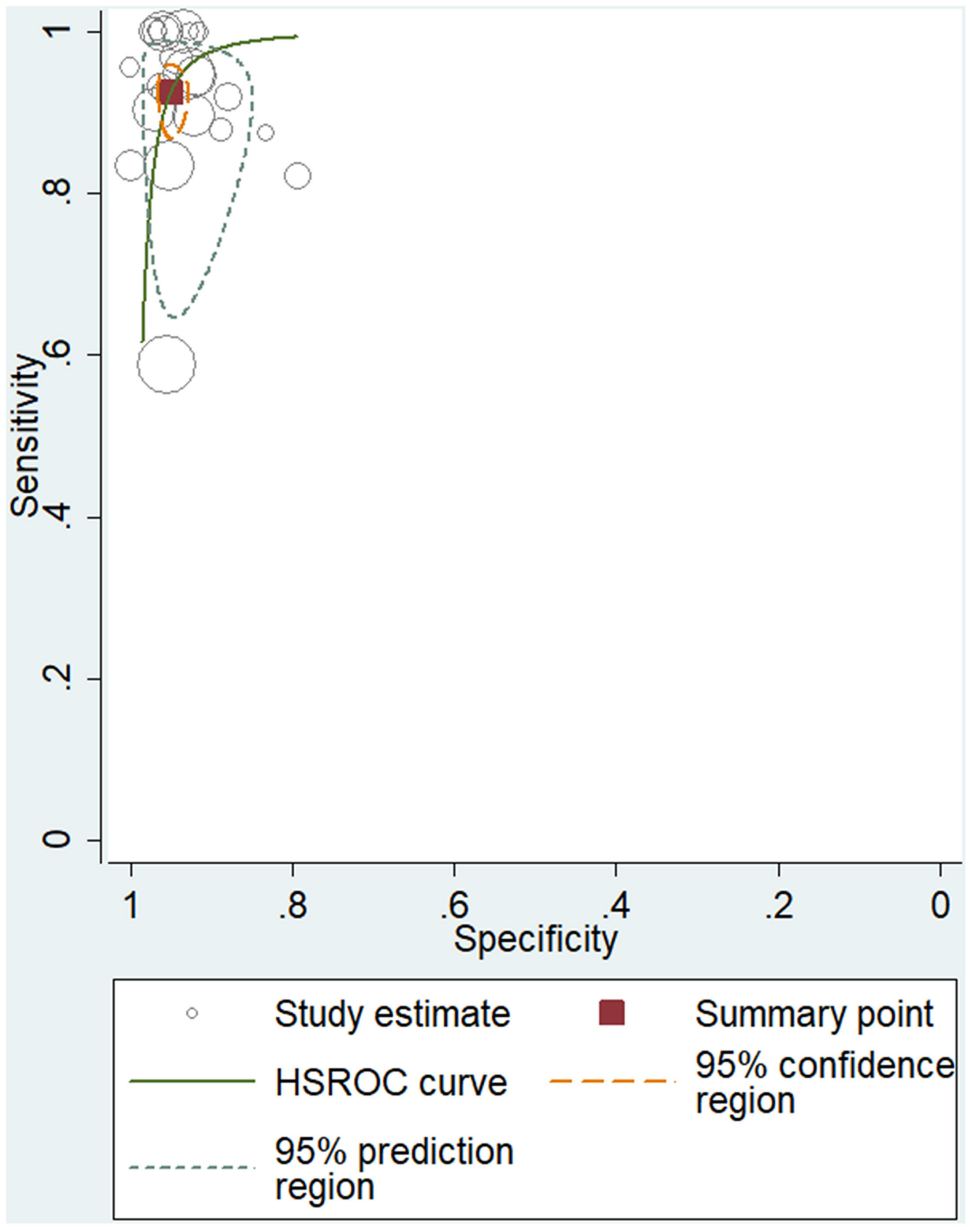
Hierarchical summary receiver operating characteristic (HSROC) curve for evaluating the overall diagnostic performance of ADA for the diagnosis of abdominal TB.

To study the summary diagnostic value of ADA for abdominal TB, 3,044 samples of 24 studies were included. The pooled sensitivity and specificity of ADA were 93% (95% CI: 0.89–0.95) and 95% (95% CI: 0.93–0.96), respectively (Figure 3). The combined PLR was 18.6 (95% CI: 14.0–24.6) and NLR was 0.08 (95% CI: 0.05–0.12) (Figure 4). The combined DOR was 236 (95% CI: 134–415), indicating that ADA was reliable for diagnosing abdominal TB. The HSROC curve of ADA with its confidence and prediction regions is shown in Figure 5. The summary point is the optimal combination of sensitivity and specificity. The yellow dotted line around each summary point represents the 95% CI. The AUROC was 0.98 (95% CI: 0.96–0.99), suggesting that ADA had excellent diagnostic accuracy (AUROC above 0.93 was considered “excellent”) (63).

### Heterogeneity

We also explored whether there was heterogeneity among potential covariates or not (Table 3). There was no heterogeneity found between the abdominal TB and control groups in the four subgroups (all *p* > 0.05): high-burden vs. low-burden TB countries, *p* = 0.12; case-control vs. cohort and cross-sectional studies, *p* = 0.35; TB ascites vs. peritoneal TB and tuberculous peritonitis, *p* = 0.11; and a cut-off value of ADA ≥40 IU/L vs. <40 IU/L, *p* = 0.26.

**Table 3:** Analysis of heterogeneity sources.

	**Covariate**	**Studies**	**Sensitivity (95%)**	**Specificity (95%)**	***p*-value**
TB burden	High	15	0.94 [0.91–0.97]	0.95 [0.93–0.96]	0.12
	Low	9	0.87 [0.81–0.94]	0.95 [0.93–0.97]	
Study design	Case-control	2	0.85 [0.70–1.00]	0.94 [0.91–0.98]	0.35
	Cohort and Cross-sectional	22	0.93 [0.90–0.96]	0.95 [0.94–0.97]	
Category	TB ascites	2	0.99 [0.96–1.00]	0.95 [0.91–1.00]	0.11
	Peritoneal TB and Tuberculous peritonitis	22	0.91 [0.88–0.95]	0.95 [0.94–0.96]	
ADA cut-off value	≥40 IU/L	4	0.96 [0.92–1.00]	0.96 [0.94–0.99]	0.26
	< 40 IU/L	20	0.91 [0.88–0.95]	0.95 [0.93–0.96]	

TB, tuberculosis.

### Publication bias

**Figure 6:**
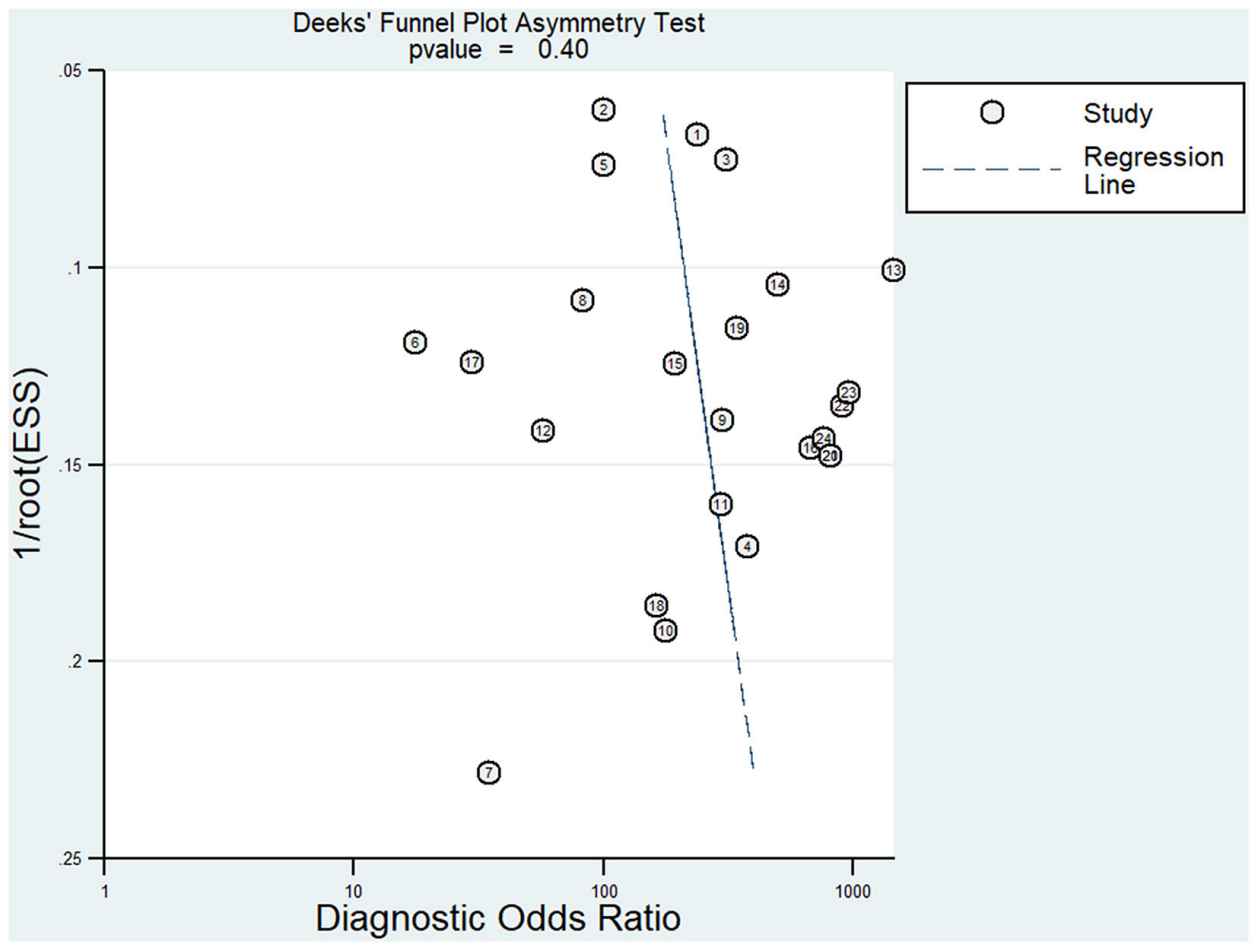
Deek's funnel plot asymmetry test for identifying publication bias.

According to Deeks' funnel plot asymmetry test (Figure 6), significant publication bias (*p* = 0.40) was not observed in any of the included articles. Therefore, the stability of this study was confirmed.

## Discussion

Abdominal TB is a disease of an insidious nature with non-specific clinical features (64). Early differentiation from other diseases and diagnosis of abdominal TB is key to successful treatment thereof (65–67). Traditional diagnostic methods can result in significant delays in the diagnosis of abdominal TB. Subsequently, severe sequelae may occur due to delayed initiation of treatment. Therefore, it is important to develop a simple, fast, and economical method to diagnose abdominal TB. Studies have found that the level of immunomodulatory enzyme ADA increases rapidly and may therefore be useful for the detection of pulmonary and/or extrapulmonary TB. However, there are no systematic studies on the diagnostic performance of ADA for abdominal TB (23, 31, 68). Hence, we performed a systematic review and meta-analysis consisting of 24 studies to assess the overall performance of ADA in abdominal TB diagnosis.

First, we evaluated the diagnostic efficacy of ADA for abdominal TB, and found the pooled sensitivity and specificity were 93 and 95%, respectively, which demonstrates that the missed diagnosis and misdiagnosis rates of abdominal TB using ADA have been as low as 7 and 5%, respectively. These findings were similar to those of ADA sensitivity and specificity for the detection of TB ascites or tuberculous peritonitis, both of which have high diagnostic efficacy (69–71). As the sensitivity and specificity were higher than 90%, the diagnostic accuracy of ADA for abdominal TB was quite high. In addition, higher than 10 of PLR values and lower than 0.1 of NLR values are considered strong diagnostic significance (72). In our meta-analysis, the PLR was 18.6, indicating that the probability of an ADA-positive diagnosis of abdominal TB was 18.6-fold higher than that of non-TB controls. Furthermore, the NLR was 0.08, suggesting that 8% of ADA-negative diagnoses were abdominal TB. DOR is a measure of diagnostic test efficiency that combines sensitivity and specificity; a higher DOR value indicates better performance of the discriminatory test (73). In this study, the DOR was 236, indicating that ADA is a good marker to distinguish abdominal TB from non-TB groups. The HSROC curve also suggested that ADA had an excellent performance in diagnosing abdominal TB; the AUROC reached 0.98, which represents a high overall accuracy with high values of sensitivity and specificity. Therefore, ADA be an accurate marker to support the diagnosis of abdominal TB, however, it cannot be used as the only diagnostic marker of abdominal TB as it has a misdiagnosis rate of 5%. In addition, ADA sensitivity was found to be as low as 58.8–83.3% in low-burden countries such as the USA, South Korea, and Spain (29, 30, 56). As a screening marker for abdominal TB, the cut-off value of ADA should be reduced in these low-burden countries to increase its sensitivity.

After evaluating the comprehensive diagnostic efficacy of ADA, bivariate analysis was carried out on TB burden, study design, category, and ADA cut-off value. No significant differences were found between these four categories (*p* > 0.05). As for TB burden, ADA did not bias the diagnosis of abdominal TB in high- and low-burden countries, although in the included studies, sensitivity was lower in low-burden countries than in high-burden countries. We also found no bias relating to study design, indicating that the original case-control studies we included did not reduce the quality of our meta-analysis. Different categories of abdominal TB did not lead to bias in the diagnostic performance of ADA, which was consistent with previous findings that ADA has superior diagnostic performance for TB ascites and tuberculous peritonitis (69, 70). Although different ADA cut-off values were reported in different original studies (7–41.1 IU/L), this wide range of values did not lead to bias in diagnosing abdominal TB. Recently, 40 IU/L was identified as the clinical diagnostic point in some studies (26, 40). As there was no bias in TB burden, study design, category, and ADA cut-off value, the results of this study are highly accurate.

Although we found that ADA had excellent diagnostic efficacy for abdominal TB without significant heterogeneity or publication bias, its limitations cannot be ignored. First, the combined sensitivity and specificity of ADA were very high (>90%). However, these two values were directly related to the prevalence of abdominal TB. As the positive predictive value of ADA increases with high prevalence, it is more important to diagnose abdominal TB using ADA in countries with high burdens of TB (74). Second, although the good values of PLR and NLR proved the diagnostic accuracy of ADA, missed diagnosis and misdiagnosis rates existed (<10%). Therefore, ADA cannot be used as the golden standard for the detection of abdominal TB. Third, ADA levels could be affected by other factors. For example, Delacour et al. (75) found that bilirubin > 50 μmol/L or hemoglobin > 177 μmol/L interfered with ADA values. In one of the studies included, Dahale et al. investigated the diagnostic value of ADA for peritoneal TB in cirrhosis. The ADA cut-off value of peritoneal TB in the cirrhosis subgroup (64.0 IU/L) was slightly lower than that of the peritoneal TB group (72.2 IU/L), which might be related to the interference of bilirubin changes in cirrhosis on ADA value (42). Fourth, HIV-induced immunodeficiency increases the likelihood of *Mtb* infection, and patients living with HIV have lower ADA levels than their seronegative counterparts (76). However, the articles included in this meta-analysis could not provide data for studying the impact of HIV infection on ADA diagnosis of abdominal TB. Finally, in the present meta-analysis, we only included English-written articles, and it is unclear whether the non-English articles would affect the results.

## Conclusions

In conclusion, this study showed that ADA has excellent diagnostic value for abdominal TB, with high sensitivity and specificity, particularly in regions with a high burden of TB. ADA detection is a simple, fast, and economical auxiliary method for the clinical diagnosis of abdominal TB.

## Data availability statement

The original contributions presented in the study are included in the article/supplementary material, further inquiries can be directed to the corresponding authors.

## Author contributions

RZ, XQ, JY, and DM conceived the study, which was refined by YY, TR, LY, QL, XS, SW, YQ, and XL. RZ and XQ conducted the literature search. RZ, XQ, JY, YY, and TR screened the full-text papers and extracted the data. RZ, XQ, TR, and SW run the analysis. RZ, XQ, JY, QL, XS, YQ, XL, and DM drafted the manuscript. All authors provided input into revisions and approved the final draft for submission.

## Funding

This work was supported by the National Natural Science Foundation of China (81801629, 81971433, 81971428, and 82071353); the National Key R&D Program of China (2021YFC2701700 and 2017YFA0104200); the grants from the Science and Technology Bureau of Sichuan Province (2021YJ0017 and 2020YFS0041); the Fundamental Research Funds for the Central University (SCU2021D009).

## Conflict of interest

The authors declare that the research was conducted in the absence of any commercial or financial relationships that could be construed as a potential conflict of interest.

## Publisher's note

All claims expressed in this article are solely those of the authors and do not necessarily represent those of their affiliated organizations, or those of the publisher, the editors and the reviewers. Any product that may be evaluated in this article, or claim that may be made by its manufacturer, is not guaranteed or endorsed by the publisher.

## Abbreviations

ADA: adenosine deaminase
AUROC: area under the receiver operating characteristic curve
CI: confidence interval
DOR: diagnostic odds ratio
FN: false negative
FP: false positive
HSROC: hierarchical summary receiver operating characteristic
HIV: human immunodeficiency virus
*M. tb*: *Mycobacterium tuberculosis*
NLR: negative likelihood ratio
PLR: positive likelihood ratio
QUADAS-2: Quality Assessment of Diagnostic Accuracy Studies tool-2
TB: tuberculosis
TN: true negative
TP: true positive.
